# Unplanned health service utilisation among people living with cancer and mental health conditions: A retrospective matched case-comparison study in Australia

**DOI:** 10.1177/00048674261470996

**Published:** 2026-08-10

**Authors:** Ramya Walsan, Reema Harrison, Huah S. Ng, Eng-Siew Koh, Sabe Sabesan, Ashfaq Chauhan, Anita Vandyke, Natalie Taylor, Elizabeth Manias, Tim Badgery-Parker, Rebecca Mitchell

**Affiliations:** 1 Australian Institute of Health Innovation, Faculty of Medicine, Health and Human Sciences, Macquarie University, North Ryde, NSW, Australia; 2 Flinders Health and Medical Research Institute, College of Medicine and Public Health, Flinders University, Adelaide, SA, Australia; 3 Collaboration for Cancer Outcomes Research and Evaluation, Ingham Institute for Applied Medical Research, South Western Sydney Clinical School, UNSW Sydney, Liverpool, Australia; 4 Department of Radiation Oncology, South West Sydney Local Health District, Liverpool, NSW, Australia; 5 Townsville Cancer Centre, University Hospital, Townsville, QLD, Australia; 6 Health Infrastructure NSW, St Leonards, Australia; 7 Faculty of Medicine and Health, University of New South Wales, Sydney, Australia; 8 Monash Nursing and Midwifery, Monash University, Clayton, VIC, Australia

**Keywords:** Cancer, mental health, unplanned service use, emergency department visits, hospitalisations, virtual care

## Abstract

**Background:**

Mental health conditions are associated with inequities across the cancer pathway, yet differences in unplanned health service use remain poorly described. This study examined sociodemographic, clinical and virtual care use factors associated with unplanned health service use among adults living with cancer, with and without mental health conditions.

**Methods:**

A retrospective matched case-comparison study was conducted using linked data from three Australian states. Adults aged ⩾ 18 years with cancer were included if they had an outpatient oncology visit between 2018 and 2021 and had either an inpatient or ambulatory mental health encounter. Individuals with mental health conditions were matched 1:1 to individuals without using propensity scores. Adjusted negative binomial models estimated incidence rate ratios for unplanned emergency department use, low-acuity emergency department use, unplanned hospitalisations and cancer-related hospitalisations within 12 months.

**Results:**

Among 99,230 adults with cancer, 9925 (10%) had a recorded mental health condition. After matching, individuals with mental health conditions had higher rates of unplanned emergency department use (incidence rate ratio = 1.34, 95% confidence interval = [1.30–1.39]), low-acuity emergency department use (incidence rate ratio = 1.26, 95% confidence interval = [1.18–1.34]), unplanned hospitalisations (incidence rate ratio = 1.32, 95% confidence interval = [1.28–1.37]) and cancer-related hospitalisations (incidence rate ratio = 1.48, 95% confidence interval = [1.41–1.55]) than those without. Virtual care use, digestive and lung cancers (vs breast), and ⩾ 1 Charlson comorbidity were also associated with increased unplanned use.

**Conclusions:**

Coexisting mental health conditions are linked to greater unplanned service use among people with cancer. Integrating mental health support into oncology care and coordinated models may reduce disparities. Further research should clarify the role of virtual care in this population.

## Introduction

People living with mental health conditions experience persistent inequities across the cancer care pathway (Grassi and Riba, 2021; Grassi et al., 2025). Compared with the general population, individuals with mental health conditions are less likely to participate in cancer screening (Benitez Majano et al., 2022), more likely to present with advanced-stage cancer (Grassi et al., 2025) and less likely to receive guideline-concordant treatment (Wang et al., 2023), leading to higher rates of invasive procedures (Wang et al., 2023) and preventable cancer-related mortality (Lin et al., 2016). Structural and interpersonal barriers, including limited social support, communication barriers and cognitive impairment, along with reduced adherence and limited insight, may further compromise engagement with healthcare and increase the risk of treatment complications and poorer outcomes (Grassi et al., 2025). Importantly, the relationship between cancer and mental health is bidirectional, as a cancer diagnosis and its treatment can increase exposure to psychological distress and the onset of symptoms of anxiety or depression or exacerbation of existing mental health conditions (Grassi and Riba, 2020).

While barriers to care and poorer outcomes among people living with cancer and comorbid mental health conditions are well documented, the extent to which these inequities translate into patterns of unplanned health service use has received comparatively less attention. Unplanned health care during cancer treatment, including emergency department (ED) presentations and hospital admissions, has been consistently associated with poorer patient outcomes (Wei et al., 2023; Yilmaz et al., 2025) and often signals treatment-related complications, unmet care needs or gaps in routine oncology care (Handley et al., 2018). Such unplanned care can place a strain on hospital systems and contributes to potentially avoidable healthcare costs (Mahumud et al., 2025). One study from the United States reported higher rates of all ED visits and hospitalisations among cancer patients with anxiety and depression (Mausbach et al., 2020). Similarly, a recent Australian study reported an association between mental health comorbidity and unplanned acute care use among people living with cancer (Walsan et al., 2026a). However, both analyses relied on regression-based approaches without a comparison or control group, and mental health status was not the primary exposure of interest in the Australian study. The lack of studies using robust comparative designs limits the ability to isolate the independent contribution of mental health conditions from underlying sociodemographic and clinical differences. Further studies employing comparison populations are needed to better characterise patterns of unplanned service use related to mental health conditions and to identify associated patient factors to inform targeted interventions for people living with cancer and mental health conditions.

The limited understanding of patterns and drivers of unplanned health service use in this population is particularly important in the context of evolving cancer care delivery models and increasing reliance on outpatient and virtual care (e.g. delivery of healthcare services remotely such as via video conferencing, phone consultations and app-based monitoring) following the COVID-19 pandemic (Williamson, 2008). While virtual care offers opportunities to improve care coordination and support the integration of oncology and mental health services, its association with unplanned health service use among people living with cancer and mental health conditions remains poorly understood. Previous Australian research has reported that use of virtual oncology services was associated with higher unplanned acute care use among patients with cancer (Walsan et al., 2026b). Individuals living with cancer and mental health conditions may be particularly vulnerable in virtual care contexts, where challenges in symptom recognition, communication and self-advocacy may increase the risk of complications and subsequent unplanned acute care (Butt et al., 2022). Clarifying the association between virtual care use and unplanned service use in this population is therefore critical in contemporary cancer care delivery. Accordingly, this study used a matched case-comparison population-based design to explore sociodemographic, clinical and virtual care use characteristics associated with unplanned health service use among people living with cancer, with and without mental health conditions, using administrative data from New South Wales (NSW), Queensland and Victoria, Australia.

## Methods

A retrospective cohort case-comparison study was conducted using linked administrative health data from public outpatient oncology services in NSW, Victoria and Queensland, Australia, the three most populous states in the country. In Australia, cancer care is delivered across both public and private settings; however, the majority of care, approximately 70–80%, occurs within the public system (McNeill et al., 2021; Pham et al., 2026). Ethical approval and a waiver of consent were obtained from the NSW Population Health Services Research Ethics Committee (2024/ETH01545) and the Victorian Department of Health and the Department of Families, Fairness and Housing Human Research Ethics Committee (HREC/97793/DOH-2023-383794(v3)). Victorian ethical approval was accepted by Queensland Health under Section 282 of the Public Health Act 2005 (PHA 97793).

### Data sources

Outpatient oncology use data from the NSW non-admitted patient data collection (NSW NAP), the Victorian Integrated Non-Admitted Health minimum dataset (VINAH) and the Queensland Health Non-Admitted Patient Data Collection (QHNAPDC) were linked to ED presentations, cancer registries, ambulatory hospital-based mental health datasets, hospital admissions and mortality datasets in each state. NSW NAP, VINAH and QHNAPDC capture all public outpatient services in NSW, Victoria and Queensland, including patient demographics, service dates, service types, contact streams and delivery modes. Outpatient oncology service use was identified in NSW NAP and QHNAPDC using Tier 2 clinic codes for oncology, medical oncology consultation, chemotherapy consultation, and radiation oncology consultation, simulation and planning. In VINAH, outpatient oncology service use was identified using contact stream ID 110 (oncology services). The cancer type was obtained from the NSW, Victorian and Queensland Cancer Registries, which are population-based registries receiving notifications on all cancer diagnoses except non-melanoma skin cancers. Cancer morphology data were available only from the Victorian and Queensland Cancer Registries, while information on degree of spread was available only from the NSW Cancer Registry.

The ED presentations were identified from the NSW Emergency Department Data Collection, the Victorian Emergency Minimum Dataset and the Queensland Emergency Data Collection, which capture patient demographics, presentation details and visit types to public hospitals. Hospital admissions and comorbidities, including mental health conditions, were obtained from diagnosis classifications in the NSW Admitted Patient Data Collection, Victorian Admitted Episodes Dataset and Queensland Hospital Admitted Patient Data Collection, covering admissions to both public and private hospitals classified using the International Classification of Diseases 10 Australian Modification (ICD-10-AM).

Additional information on mental health was obtained from the NSW Ambulatory Mental Health Data Collection, the Client Management Interface/Operational Data Store in Victoria and the Consumer Integrated Mental Health and Addiction system in Queensland, which capture state-wide public mental health, alcohol and other drug service use. Mortality data, including fact and date of death, were sourced from the respective Registries of Births, Deaths and Marriages. Data were probabilistically linked by the Centre for Health Record Linkage, the Centre for Victorian Data Linkage and the Queensland Data Services Branch.

### Case inclusion criteria

Cases included adults aged ⩾ 18 years residing in NSW, Victoria or Queensland who received outpatient oncology services between 1 January 2018 and 31 December 2020, had a cancer diagnosis recorded in a state cancer registry (ICD-10-AM codes: C00–C43, C45–C96, D45, D46, D47.1, D47.3–D47.5) and had at least one hospital admission or outpatient mental health service encounter with a recorded mental health diagnosis during the study period. Participants were followed for 12 months from their index outpatient oncology encounter in the study period or censored at death.

Oncology service use was identified using contact stream ID 110 in VINAH and Tier 2 clinic codes for oncology, medical oncology consultation, chemotherapy and radiation oncology (consultation, simulation and planning) in NSWNAP and QHNAPDC. Mental health conditions were identified from hospital admission and outpatient mental health records using ICD-10-AM codes (F00–F99). Serious mental illness (SMI) included schizophrenia spectrum and related disorders (ICD-10-AM: F20–F29) and bipolar affective disorders (ICD-10-AM: F30–F31) (Mansour et al., 2020); depression included major depressive disorders (ICD-10-AM: F32–F39) (Lee et al., 2021); and anxiety disorders (ICD-10-AM: F40–F43) (Chai et al., 2024). Other mental disorders comprised the remaining ICD-10-AM mental and behavioural disorder codes. Mental health condition categories were used to characterise the cohort descriptively and were not included in the propensity score matching or post-matching analytical models, as the primary exposure was the presence versus absence of any mental health condition.

### Comparison inclusion criteria

The comparison cohort included adults aged ⩾ 18 years residing in NSW, Victoria or Queensland who received outpatient oncology services between 1 January 2018 and 31 December 2020 and had a cancer diagnosis recorded in a cancer registry, with no recorded mental health diagnosis in hospital admission or outpatient mental health records during the study period. The comparison cohort was also followed for 12 months from their index encounter within the study period or censored at death.

### Identification of comorbidities and clinical characteristics

Cancer type was identified using primary site ICD-10-AM codes from the cancer registries, with inpatient records used where necessary. The four most common cancers, breast, digestive (including colorectal), lung and prostate, were analysed separately, while less common cancers were grouped (Supplementary Table 1). Cancer morphology and degree of spread were reported descriptively but excluded from analysis due to absence of data across states. Comorbidities were identified from admission diagnoses during the study period using the 17 Charlson comorbidities, excluding malignancy to avoid collinearity with the cancer diagnosis, and were classified as present or absent. Virtual care was defined as outpatient oncology consultations delivered by telephone or video, based on the mode of delivery within 12 months from the index encounter in the study period. To account for prior outpatient health service use, individuals were categorised according to their outpatient activity in the year before the study period (1 January–31 December 2017). Both virtual care use during follow-up and pre-index outpatient use were classified based on the number of encounters into no use, low use (1–3 encounters) or high use (⩾ 4 encounters) (Soril et al., 2016).

### Sociodemographic characteristics

Patients’ residential location in the outpatient data was classified using Statistical Area Level 2 (SA2) in NSW and Queensland and postcode in Victoria and mapped to the Australian Statistical Geography Standard (ASGS) Remoteness classification (Australian Bureau of Statistics, 2016a). The five remoteness categories were collapsed into two groups: urban (major cities) and rural (inner regional, outer regional, remote and very remote). Socioeconomic disadvantage was estimated using the Index of Relative Socioeconomic Disadvantage (IRSD) (Australian Bureau of Statistics, 2018), assigned from each patient’s SA2 or postcode of residence and grouped into quintiles (Q1 = most disadvantaged; Q5 = least disadvantaged). Country of birth was classified using the Standard Australian Classification of Countries (Australian Bureau of Statistics, 2016b) and identified as Australian- or overseas-born using inpatient or outpatient records. Patients whose 12-month follow-up period overlapped with the major COVID-19 lockdowns in three states (1 March 2020 to 31 July 2021) were also identified.

### Study outcomes

Outcomes included counts of unplanned ED visits, unplanned low-acuity ED visits, unplanned hospitalisations and unplanned hospitalisations with a principal diagnosis of cancer within 12 months of the index encounter. Unplanned hospitalisations with a principal diagnosis of mental illness were not included due to small numbers. Unplanned ED visits were defined as presentations with a visit type of ‘emergency presentation’. Unplanned low-acuity ED visits were identified using a proxy of triage categories 4 and 5 (semi-urgent or non-urgent) (Dinh et al., 2016). Unplanned hospitalisations were defined as admissions with a status of ‘emergency admission’.

### Data management and analysis

Data were analysed using SAS version 9.4 (SAS Institute Inc.) within the Secure Unified Research Environment (SURE). Imbalances in potential confounders between patients with cancer with and without a mental health comorbidity were addressed using propensity score case-comparison matching. Propensity scores, representing the predicted probability of having a mental health comorbidity among patients with cancer, were estimated using multivariable logistic regression. The propensity score model included age group, sex, country of birth, Charlson comorbidities excluding malignancy, residential remoteness, socioeconomic status, COVID-19 lockdown period, cancer type, and pre-index outpatient and virtual care use as covariates.

Case-comparison 1:1 greedy nearest-neighbour propensity score matching without replacement was performed on the logit of the propensity score, using a calliper width of 0.2 standard deviations (Austin, 2011, 2014). Patients with a recorded mental health comorbidity were matched to patients without a recorded mental health comorbidity only when a suitable comparator was available within the specified calliper. Otherwise, they were excluded from the matched cohort. Covariate balance before and after matching was assessed using standardised mean differences (SMD), with values < 0.10 indicating adequate balance. Of 9925 patients with a mental health comorbidity in the original cohort, 9270 (93.4%) were successfully matched to patients without a mental health comorbidity, yielding a matched cohort of 18,540 patients. A total of 80,690 cases did not have an identified match. Among these, 57,668 (66%) were aged 45–65 years, 40,659 (50.4%) were females, 51,267 (63.6%) resided in urban areas and 34,430 (42.7%) were in the two most disadvantaged socioeconomic quintiles. In addition, 15,019 (32.9%) had breast cancer.

Descriptive statistics (counts and percentages) were used to summarise demographic and clinical characteristics of patients with cancer with and without a mental health comorbidity before and after matching. Negative binomial regression models were fitted after propensity score matching to estimate adjusted associations in the matched cohort and account for remaining measured differences after matching (Lindenauer et al., 2005; Nguyen et al., 2017). Models examined factors associated with unplanned ED visits, low-acuity ED visits, and unplanned hospitalisations and hospitalisations due to cancer during the 12-month follow-up period. Covariates were selected a priori based on known associations with healthcare utilisation (Abdel-Rahman, 2021; Handley et al., 2018; Lash et al., 2017) and data availability and included mental health comorbidity status, age group, sex, country of birth, number of Charlson comorbidities (excluding malignancy), residential remoteness, socioeconomic status, cancer type, COVID-19 lockdown period, pre-index outpatient use and virtual care use. Propensity score matching and subsequent analyses were conducted using complete-case data. No significant interaction effects were observed. Results are presented as incidence rate ratios (IRRs) with 95% confidence intervals (CIs).

To assess the potential impact of immortal time bias or time-alignment bias due to ascertainment of diagnoses after cohort entry, a sensitivity analysis using a person-specific 12-month pre-index lookback period to define mental health comorbidity was conducted. In this analysis, the index date remained the first eligible outpatient oncology encounter and defined the start of the 12-month outcome follow-up period. Patients were classified as having a mental health comorbidity only if a mental health diagnosis was recorded in hospital admission or outpatient mental health records during the 12 months before the index date. Charlson comorbidities were also defined using only hospital admission diagnoses recorded during the pre-index period. Propensity score matching and negative binomial regression models were repeated using the same approach as in the primary analysis.

## Results

The study included 99,230 people living with cancer, of whom 9925 (10.0%) had a recorded mental health condition. After propensity score matching, 9270 pairs of patients with and without a mental health comorbidity were identified, yielding a matched cohort of 18,540 patients. Among patients with a mental health comorbidity, over half (55.1%) were aged 65–74 years, 56.4% were male, 63.4% resided in urban areas and 40.7% had at least one Charlson comorbidity. Most patients were born in Australia (72.3%), and a substantial proportion lived in socioeconomically disadvantaged areas, with 27.9% residing in the most disadvantaged quintile. Lung (39.0%) and digestive system cancers (37.1%) were the most common cancer types. Approximately one-quarter (25.5%) of patients had high virtual outpatient use (⩾ 4 visits per year), while 16.3% had high pre-index outpatient utilisation. Around 40.3% of patients experienced care during a COVID-19 lockdown period (Table 1). Among patients with a mental health comorbidity, anxiety-related disorders, depression and all SMIs accounted for 18.3%, 14.0% and 10.7% of diagnoses, respectively.

**Table 1.** Baseline demographic and clinical characteristics of patients with cancer with and without a mental health comorbidity before and after propensity score matching, New South Wales, Queensland and Victoria, Australia, 2018-2021.

Characteristics	Before matching					After matching				
	Cancer with mental health comorbidity (*n* = 9925; 10%)		Cancer without mental health comorbidity (*n* = 89,305; 90.0%)			Cancer with mental health comorbidity (*n* = 9270; 50%)		Cancer without mental health comorbidity (*n* = 9270; 50%)		
	n	%	n	%	SMD^a^	n	%	n	%	SMD^a^
Age categories										
18–44 years	692	7.5	8026	9.0	0.01	692	7.4	643	6.9	0.00
45–64 years	3476	37.5	32,126	36.1		3474	37.5	3446	37.2	
65–74 years	2740	29.5	26,711	30.1		2739	29.6	2726	29.4	
⩾ 75 years	2366	25.5	22,156	24.9		2365	25.5	2455	26.5	
Gender										
Male	5505	55.5	45,038	50.4	0.11	5231	56.4	5293	57.1	0.00
Female	4418	44.5	44,257	49.6		4039	43.6	3977	42.9	
Country of birth										
Australia	7164	72.6	60,382	68.2	0.10	6665	72.3	6680	72.5	0.00
Other	2698	27.4	28,084	31.8		2557	27.7	2537	27.5	
Rurality										
Urban	6324	63.8	56,706	63.5	0.00	5875	63.4	5888	63.5	0.00
Rural	3595	36.2	32,577	36.5		3392	36.6	3379	36.5	
Socioeconomic status										
Q1 (Most disadvantaged)	2710	27.4	20,637	23.1	0.16	2578	27.9	2568	27.8	0.00
Q2	2191	22.1	18,261	20.5		2092	22.6	2131	23.0	
Q3	1935	19.6	17,855	20.0		1815	19.6	1790	19.3	
Q4	1634	16.5	16,570	18.6		1499	16.2	1559	16.9	
Q5 (Least disadvantaged)	1429	14.4	15,864	17.8		1263	13.7	1206	13.0	
Charlson comorbidities (excluding malignancy)										
No	4315	59.3	60,650	74.5	0.51	4314	59.3	4281	58.7	0.01
Yes	2958	40.7	20,710	25.5		2958	40.7	3017	41.3	
Cancer type										
Breast	895	15.3	15,675	31.1	0.01	784	14.7	767	14.6	0.00
Digestive organs, including colorectal	2078	35.5	15,359	30.5		1976	37.1	2014	38.4	
Lung	2150	36.7	9155	18.2		2079	39.0	2016	38.4	
Prostate	268	4.6	4483	8.9		238	4.5	227	4.3	
Other cancers/unknown primary site/ill-defined	467	7.8	5677	11.3		256	4.8	222	4.2	
Cancer morphology^b^										
Well or moderately differentiated	1559	26.1	13,207	28.3	0.00	1388	15.0	1047	11.3	0.00
Poorly or undifferentiated	1366	22.8	10,670	22.8		1236	13.3	870	9.4	
Unspecified/missing	3058	51.1	22,856	51.1		6646	71.7	7353	79.3	
Degree of cancer spread^c^										
In situ/localised	1016	25.8	14,786	34.7		1013	25.7	1450	26.8	
Regionalised	897	22.8	9891	23.2		897	22.8	1225	22.6	
Metastatic	1111	28.2	8126	19.1		1111	28.2	1639	30.3	
Unknown	918	23.3	9769	23.0		918	23.3	1103	20.4	
Mental health condition type^d^										
Serious mental illness ^e^	1307	13.2	–	–	–	996	10.7	–	–	–
Anxiety	1895	19.1	–	–		1693	18.3	–	–	
Depression	1718	17.3	–	–		1294	14.0	–	–	
Others	5005	50.4	–	–		5287	57.0	–	–	
Virtual outpatient use										
No use	4310	43.4	39,628	44.4	0.03	3914	42.2	4110	44.3	0.08
Low use (⩽ 3 use/year)	3123	31.5	26,516	29.7		2988	32.2	3059	33.0	
High use (⩾ 4 use/year)	2492	25.1	23,161	25.9		2368	25.5	2101	22.7	
Pre-index outpatient use										
No use/low use	8177	82.4	73,038	81.8	0.04	7757	83.7	7836	84.5	0.00
High use (⩾ 3/year)	1748	17.6	16,267	18.2		1513	16.3	1434	15.5	
COVID-19 lockdown										
No	6087	61.3	50,848	56.9	0.05	5531	59.7	5463	58.9	0.00
Yes	3838	38.7	38,457	43.1		3739	40.3	3807	41.1	

a Standardised mean difference.

b Cancer morphology data were available only from the Queensland and Victorian Cancer Registries (*n* = 52,716) and were therefore not included in propensity score matching due to the absence of comparable data from New South Wales.

c Cancer stage at diagnosis was available only from the New South Wales Cancer Registry (*n* = 46,514) and was not included in propensity score matching due to the absence of comparable data from Queensland and Victoria.

d Mental health condition types are presented for descriptive purposes only and were not included in propensity score matching because mental health status was the primary exposure variable.

e Serious mental illness included schizophrenia spectrum and related disorders and bipolar affective disorders.

In the propensity score-matched cohort, patients with a mental health comorbidity had a higher proportion of at least one unplanned ED visit compared to patients without mental health conditions (53.7% vs 46.3%) and a higher mean number of unplanned ED visits (mean [SD] = 2.46 [3.07] vs 1.83 [2.40]). Similar patterns were observed for low-acuity unplanned ED visits (53.4% vs 46.7%; mean [SD] = 0.64 [1.48] vs 0.49 [1.18]), unplanned hospitalisations (54.6% vs 45.4%; mean [SD] = 1.76 [2.01] vs 1.342 [1.77]) and unplanned hospitalisations with a principal diagnosis of cancer (57.4% vs 42.6%; mean [SD] = 0.57 [0.89] vs 0.39 [0.73]).

Multivariable negative binomial regression analyses conducted in the propensity score-matched cohort showed that compared with patients without a mental health comorbidity, patients with a mental health comorbidity had higher rates of unplanned ED visits (IRR = 1.34, 95% CI = [1.30–1.39]), low-acuity unplanned ED visits (IRR = 1.26, 95% CI = [1.18–1.34]), unplanned hospitalisations (IRR = 1.32, 95% CI = [1.28–1.37]) and unplanned hospitalisations with a principal diagnosis of cancer (IRR = 1.48, 95% CI = [1.41–1.55]) (Table 2).

**Table 2.** Factors associated with unplanned healthcare utilisation in the propensity score-matched cohort of patients with cancer, New South Wales, Queensland and Victoria, Australia, 2018-2021.

Characteristics	Unplanned ED visits	Low acuity unplanned ED visits	Unplanned hospitalisations	Unplanned hospitalisations due to cancer
	Adjusted IRR (95% CI)	Adjusted IRR (95% CI)	Adjusted IRR (95% CI)	Adjusted IRR (95% CI)
Mental health comorbidity				
No	1	1	1	1
Yes	1.34 (1.30–1.39)	1.26 (1.18–1.34)	1.32 (1.28–1.37)	1.48 (1.41–1.55)
Age category				
18–44 years	1	1	1	1
45–64 years	0.83 (0.77–0.89)	0.89 (0.79–1.01)	0.93 (0.86–1.00)	0.95 (0.85–1.06)
65–74 years	0.76 (0.70–0.82)	0.86 (0.76–0.97)	0.94 (0.87–1.02)	0.87 (0.76–1.00)
⩾ 75 years	0.72 (0.68–0.79)	0.98 (0.87–1.11)	0.89 (0.81–1.00)	0.85 (0.76–0.95)
Gender				
Male	1	1	1	1
Female	0.93 (0.89–0.96)	0.90 (0.84–0.96)	0.95 (0.91–0.98)	0.96 (0.92–1.01)
Country of birth				
Australia	1	1	1	1
Other	0.99 (0.95–1.03)	0.83 (0.78–0.90)	1.03 (0.99–1.08)	1.08 (1.02–1.14)
Rurality				
Urban	1	1	1	1
Rural	1.16 (1.12–1.21)	1.78 (1.67–1.90)	0.94 (0.91–0.98)	0.97 (0.91–1.02)
Socioeconomic status				
Q1 (Most disadvantaged)	1	1	1	1
Q2	0.96 (0.90–0.99)	0.88 (0.82–0.96)	0.98 (0.94–1.03)	1.01 (0.95–1.08)
Q3	0.89 (0.85–0.94)	0.76 (0.69–0.83)	0.96 (0.91–1.01)	0.96 (0.89–1.03)
Q4	0.81 (0.77–0.86)	0.71 (0.65–0.79)	0.90 (0.85–0.95)	0.91 (0.84–0.98)
Q5 (Least disadvantaged)	0.71 (0.67–0.76)	0.69 (0.62–0.76)	0.87 (0.81–0.92)	0.81 (0.74–0.88)
COVID-19 lockdown				
No	1	1	1	1
Yes	0.95 (0.92–0.99)	0.92 (0.86–0.99)	0.91 (0.87–0.94)	0.81 (0.77–0.86)
Charlson comorbidities, excluding malignancy				
No	1	1	1	1
Yes	1.26 (1.21–1.31)	1.26 (1.17–1.35)	1.26 (1.22–1.32)	1.42 (1.34–1.50)
Cancer type				
Breast	1	1	1	1
Digestive organs including colorectal	1.41 (1.30–1.53)	1.03 (0.91–1.17)	2.12 (1.95–2.33)	3.77 (3.15–4.53)
Lung	1.47 (1.36–1.60)	0.89 (0.78–1.02)	2.38 (2.19–2.61)	5.46 (4.56–6.53)
Prostate	0.91 (0.76–1.08)	0.84 (0.66–1.08)	1.30 (1.09–1.55)	2.02 (1.52–2.68)
Other cancers/unknown primary site/ill-defined	1.09 (0.94–1.27)	1.01 (0.81–1.25)	1.66 (1.43–1.94)	1.84 (1.40–2.42)
Pre-index outpatient use				
No/low use (⩽ 2/year)	1	1	1	1
High use (⩾ 3/year)	1.07 (1.02–1.13)	1.24 (1.13–1.36)	0.96 (0.91–1.02)	0.71 (0.65–0.76)
Virtual care use				
No use	1	1	1	1
Low use (⩽ 3 use/year)	1.23 (1.18–1.29)	1.21 (1.13–1.31)	1.28 (1.22–1.34)	1.19 (1.13–1.27)
High use (⩾ 4 use/year)	1.42 (1.35–1.50)	1.54 (1.40–1.68)	1.57 (1.50–1.64)	1.24 (1.16–1.32)

Other sociodemographic, clinical and service-use characteristics were also associated with unplanned healthcare utilisation in the matched cohort. Compared with patients in the most disadvantaged areas, those in the least disadvantaged areas had lower rates of unplanned ED visits (IRR = 0.71, 95% CI = [0.67–0.76]), low-acuity ED visits (IRR = 0.69, 95% CI = [0.62–0.76]), unplanned hospitalisations (IRR = 0.87, 95% CI = [0.81–0.92]) and cancer-related hospitalisations (IRR = 0.81, 95% CI = [0.74–0.88]). Patients with Charlson comorbidities had higher rates of unplanned ED visits (IRR = 1.26, 95% CI = [1.21–1.31]), low-acuity ED visits (IRR = 1.26, 95% CI = [1.17–1.35]), unplanned hospitalisations (IRR = 1.26, 95% CI = [1.22–1.35]) and cancer-related hospitalisations (IRR = 1.42, 95% CI = [1.34–1.50]) compared to patients without any. Compared with patients with breast cancer, patients with digestive and lung cancers had higher rates of unplanned ED visits, unplanned hospitalisations and cancer-related hospitalisations, with the strongest associations observed for cancer-related hospitalisations among patients with lung cancer (IRR = 5.46, 95% CI = [4.56–6.53]) and digestive cancers (IRR = 3.77, 95% CI = [3.15–4.53]). Rural residence, compared to urban, was associated with higher rates of unplanned ED visits (IRR = 1.16, 95% CI = [1.12–1.21]) and low-acuity ED visits (IRR = 1.78, 95% CI = [1.67–1.90]) but lower rates of unplanned hospitalisations (IRR = 0.94, 95% CI = [0.91–0.98]). High virtual care use was associated with higher rates of all unplanned healthcare utilisation outcomes, including unplanned ED visits (IRR = 1.42, 95% CI = [1.35–1.50]), low-acuity ED visits (IRR = 1.54, 95% CI = [1.40–1.68]), unplanned hospitalisations (IRR = 1.57, 95% CI = [1.50–1.64]) and cancer-related hospitalisations (IRR = 1.24, 95% CI = [1.16–1.32]) (Table 2).

In the pre-index ascertainment sensitivity analysis, in which mental health comorbidity and Charlson comorbidities were defined using diagnoses recorded before the index outpatient oncology encounter, findings were consistent with the primary analysis. The analytic sample was smaller, and effect estimates were slightly stronger in magnitude with wider confidence intervals (Supplementary Tables 2 and 3).

## Discussion

This study explored unplanned health service use among patients living with cancer, with and without mental health conditions. The findings revealed that, among people receiving outpatient care, the presence of a mental health comorbidity was consistently associated with higher rates of unplanned healthcare use (i.e. unplanned ED visits, low-acuity ED visits, unplanned hospitalisations and cancer-related hospitalisations) compared to individuals with cancer alone.

The observed inequities in unplanned health service use among patients with cancer and comorbid mental health conditions in the current study are consistent with the sparse existing literature in this area (Mausbach et al., 2020; Walsan et al., 2026a), as well as with evidence of heightened morbidity and healthcare use in this population more broadly (Abdalla et al., 2024; Choi et al., 2024; Purushotham et al., 2013; Sporinova et al., 2019). These associations are anticipated given the well-documented inequities faced by people living with mental health conditions across the cancer care continuum. Mental health conditions can complicate cancer symptom appraisal and self-management, potentially leading to delayed help-seeking or increased reliance on unplanned acute care services (Defty et al., 2025). In addition, communication challenges and reduced continuity of care across oncology, primary care and mental health services may further contribute to potentially avoidable and unplanned health service presentations (Grassi et al., 2025). The stronger association observed between mental health comorbidity and cancer-related unplanned hospitalisations likely reflects the greater sensitivity of cancer-specific admissions to mental health comorbidity.

Several sociodemographic, clinical and service-use characteristics were also associated with unplanned healthcare utilisation. Higher rates of unplanned healthcare use were observed among patients with Charlson comorbidities and among those with lung and digestive system cancers, consistent with greater clinical complexity in these groups (Sánchez et al., 2023; Sarfati et al., 2016; Weidner et al., 2018). Socioeconomic disadvantage and rural residence were also associated with higher ED use, which may reflect differences in access to timely primary, specialist and supportive care (Bhatia et al., 2022; Kirby and Kaneda, 2005).

High virtual outpatient care use was associated with higher rates of unplanned healthcare utilisation across all outcomes, consistent with previous Australian findings among people with cancer (Walsan et al., 2026a, 2026b). This association may reflect limitations in virtual assessment where in-person examination or investigations are clinically important (Waseem et al., 2022) or confounding by indication, whereby patients with greater clinical complexity are both more likely to receive frequent virtual care and more likely to require acute care (Kyriacou and Lewis, 2016). Further research is needed to examine the timing, content and purpose of virtual encounters and their relationship with subsequent acute care pathways.

Overall, these findings highlight the need for earlier identification and proactive management of mental health conditions among people receiving cancer care. Targeted models of care that integrate mental health, cancer treatment planning and delivery and social support may be required to reduce disparities and improve outcomes for this population (Corveleyn et al., 2025; Fløe et al., 2025). Virtual oncology care may improve access and continuity but should be paired with psychosocial support, safe symptom triage and timely escalation to in-person care when needed for patients living with cancer and mental health conditions. The observed association between high virtual care use and unplanned healthcare utilisation warrants further investigation to clarify its role within cancer care pathways.

A key strength of this study is the use of large, population-level data from the three most populous Australian states and propensity score matching to create a well-balanced comparison cohort. However, several limitations should be noted. As an observational study, causal inference is limited and residual confounding from unmeasured factors may remain. Therefore, the findings should be interpreted as associations between recorded mental health comorbidity and unplanned acute health service use, rather than evidence of a causal effect. Mental health conditions were identified using hospital and ambulatory data, which may not capture conditions managed exclusively outside hospital settings. Complete lifetime mental health histories were not available and therefore, some patients with historical mental illness, but no recorded diagnosis during the available ascertainment period may have been classified in the comparison group. Similarly, physical comorbidities were limited to hospital-recorded diagnoses, likely capturing more severe cases. The primary analysis may be subject to time-alignment bias because mental health comorbidity and Charlson comorbidities were identified from diagnoses recorded during the study period, while follow-up began at the first eligible outpatient oncology encounter. However, a pre-index ascertainment sensitivity analysis produced consistent findings, suggesting that post-index diagnosis ascertainment is unlikely to fully explain the observed associations, although residual bias cannot be excluded. The absence of information on cancer stage, degree of spread, morphology and treatment types limited adjustment for disease severity and treatment intensity. Although virtual care use preceded unplanned health service use for most patients (97.2%), exposure and outcome were measured within the same follow-up window, raising the possibility of residual confounding. Use of a complete-case approach may have introduced selection bias if data were not missing at random. This study did not examine associations for each psychiatric diagnosis because the primary exposure of interest was any recorded mental health comorbidity. Future studies are warranted to examine whether unplanned healthcare utilisation differs by psychiatric diagnosis, illness severity and treatment status. Interpretation was further complicated by COVID-19-related service disruptions and rapid telehealth expansion, although the lockdown period was included as a covariate. COVID-19-related changes in health service use were accounted for using a binary indicator of whether follow-up overlapped with major lockdown periods. Because outcome data extended only to 2021, we were unable to examine longer-term post-pandemic patterns in unplanned healthcare utilisation. Finally, the content, quality, modality of virtual encounters or administrative data were not able to be validated.

## Conclusions

This study highlights disparities in unplanned health service use among patients living with both cancer and mental health conditions compared with individuals living with cancer alone. These findings underscore the importance of integrating mental health assessment and support within routine oncology care. Targeted models of care that integrate mental health, cancer treatment and social support may help reduce disparities, improve outcomes and reduce unplanned health service use. Further research is needed to clarify the role and optimal use of virtual care in cancer pathways, particularly for patients with complex clinical and psychosocial needs.

## Supplemental Material

sj-docx-1-anp-10.1177_00048674261470996 – Supplemental material for Unplanned health service utilisation among people living with cancer and mental health conditions: A retrospective matched case-comparison study in Australia Supplemental material, sj-docx-1-anp-10.1177_00048674261470996 for Unplanned health service utilisation among people living with cancer and mental health conditions: A retrospective matched case-comparison study in Australia by Ramya Walsan, Reema Harrison, Huah S. Ng, Eng-Siew Koh, Sabe Sabesan, Ashfaq Chauhan, Anita Vandyke, Natalie Taylor, Elizabeth Manias, Tim Badgery-Parker and Rebecca Mitchell in Australian & New Zealand Journal of Psychiatry

## Data Availability

The data that support the findings of this study are available from the NSW, Victorian and Queensland Department of Health. Restrictions apply to the availability of these data, which were used under licence for the current study, so they are not publicly available.
