# Supplementary material for: Unplanned health service utilisation among people living with cancer and mental health conditions: A retrospective matched case-comparison study in Australia
Source: Aust N Z J Psychiatry. 2026 Aug 10;60(10):941–51. doi: 10.1177/00048674261470996 (PMC13620385; doi:10.1177/00048674261470996)
Supplement: Supplementary file 1 [file sj-docx-1-anp-10.1177_00048674261470996.docx]

**Supplementary Figure 1: Cohort flow diagram**

***All n values represent unique individuals not service encounters.**

**Supplementary Table 1: Identification and Categorisation of Cancer types**

| Cancer type | ICD-10-AM |
| --- | --- |
| Breast | C50 |
| Digestive organs, including colorectal | C15-C17, C18-C20, C21-C26, |
| Lung | C33-C34 |
| Prostrate | C61 |
| Other cancers/ Unknown primary site/ill defined | C43, C44, C00-C14, C30-C32, C45-C49, C51-C55, C57.1-C57.9, C58.1-C58.9, C56, C57.0, C58.0, D359, C64, C67, C71, C72.8, C72.9, C81-C86, C88, C90-C96, D45, D46, C47.1, D47.3-D47.5, C80, C30-C32, C37-C39, C40-C41, C60, C62 -C63, C65-C66, C68, C69, C70, C72.0, C72.7, C73, C74, C75.0, C75.1-C75.3, C75.4-C75.9, C76, C60, C62-C63, C77-C78, C79 |

**Supplementary Table 2. Baseline demographic and clinical characteristics of patients with cancer with and without a mental health comorbidity defined using a 12-month pre-index lookback period, before and after propensity score matching, New South Wales, Queensland and Victoria, Australia, 2018–2021.**

| **Characteristics** | **Before matching** | | | | | **After matching** | | | | |
| --- | --- | --- | --- | --- | --- | --- | --- | --- | --- | --- |
|  | **Cancer with mental health comorbidity**  (n=2,860; 8%) | | **Cancer without mental health comorbidity**  (n=32,387; 92%) | | **SMD*** | **Cancer with mental health comorbidity**  (n=2,655; 50%) | | **Cancer without mental health comorbidity**  (n=2,655; 50%) | | **SMD*** |
|  | **n** | **%** | **n** | **%** |  | **n** | **%** | **n** | **%** |  |
| **Age categories** | | | | | | | | | | |
| 18-44 years | 227 | 8.5 | 2,743 | 8.5 | 0.06 | 227 | 4.3 | 216 | 8.1 | 0.00 |
| 45 – 64 years | 1015 | 38.2 | 10,958 | 34.0 |  | 1,015 | 19.1 | 1,009 | 38.0 |  |
| 65 – 74 years | 713 | 26.9 | 9,511 | 29.5 |  | 713 | 13.4 | 730 | 27.5 |  |
| ≥75 years | 700 | 26.4 | 9,031 | 28.0 |  | 700 | 13.2 | 700 | 26.4 |  |
| **Gender** | | | | | | | | | | |
| Male | 1,533 | 53.6 | 16,368 | 50.5 | 0.08 | 1,448 | 54.5 | 1,474 | 55.5 | 0.00 |
| Female | 1,326 | 46.4 | 16,017 | 49.5 |  | 1,207 | 45.5 | 1,181 | 44.5 |  |
| **Country of birth** | | | | | | | | | | |
| Australia | 2,034 | 71.5 | 21,434 | 66.5 | 0.09 | 1,878 | 71.0 | 1,882 | 71.1 | 0.00 |
| Other | 812 | 28.5 | 10,788 | 33.5 |  | 766 | 29.0 | 767 | 28.9 |  |
| **Rurality** | | | | | | | | | | |
| Urban | 1,951 | 68.2 | 21,406 | 66.1 | 0.05 | 1,812 | 68.3 | 1,819 | 68.5 | 0.00 |
| Rural | 909 | 31.8 | 10,970 | 33.9 |  | 843 | 31.7 | 836 | 31.5 |  |
| **Socio economic status** | | | | | | | | | | |
| Q1 (Most disadvantaged) | 803 | 28.2 | 7,641 | 23.7 | 0.14 | 770 | 29.1 | 758 | 28.6 | 0.00 |
| Q2 | 605 | 21.2 | 6600 | 20.4 |  | 565 | 21.3 | 572 | 21.6 |  |
| Q3 | 530 | 18.6 | 6,433 | 19.9 |  | 499 | 18.9 | 500 | 18.9 |  |
| Q4 | 459 | 16.1 | 5,851 | 18.1 |  | 413 | 15.6 | 431 | 16.3 |  |
| Q5 (Least disadvantaged) | 455 | 16.0 | 5,783 | 17.9 |  | 400 | 15.1 | 388 | 14.7 |  |
| **Charlson comorbidities (excluding malignancy)** | | | | | |  |  |  |  |  |
| No | 966 | 50.7 | 12,625 | 69.5 | 0.12 | 966 | 50.7 | 974 | 51.4 | 0.00 |
| Yes | 938 | 49.3 | 5,539 | 30.5 |  | 938 | 49.3 | 922 | 48.6 |  |
| **Cancer type** | | | | | |  |  |  |  |  |
| Breast | 260 | 16.4 | 5,928 | 34.6 | 0.09 | 225 | 15.9 | 210 | 14.8 | 0.00 |
| Digestive organs including colorectal | 580 | 36.6 | 5,645 | 32.9 |  | 543 | 38.3 | 546 | 38.5 |  |
| Lung | 592 | 37.3 | 3,463 | 20.2 |  | 565 | 39.8 | 579 | 40.8 |  |
| Prostate | 27 | 1.7 | 707 | 4.1 |  | 21 | 1.5 | 25 | 1.8 |  |
| Other cancers/ unknown primary site/ill defined | 127 | 8.0 | 1,407 | 8.2 |  | 64 | 4.5 | 58 | 4.5 |  |
| **Cancer morphology ^a^** | | | | | | | | | | |
| Well or moderately differentiated | 258 | 28.7 | 2,784 | 31.3 |  | 201 | 29.0 | 179 | 28.7 |  |
| Poorly or undifferentiated | 228 | 25.4 | 1,991 | 22.4 |  | 190 | 27.4 | 144 | 23.1 |  |
| Unspecified/Missing | 412 | 45.9 | 4,114 | 46.3 |  | 302 | 43.6 | 300 | 48.2 |  |
| **Degree of cancer spread ^b^** | | | | | | | | | | |
| Insitu/localised | 409 | 20.9 | 8,165 | 34.8 |  | 409 | 20.9 | 547 | 26.9 |  |
| Regionalised | 360 | 18.4 | 5,229 | 22.2 |  | 360 | 18.4 | 453 | 22.3 |  |
| Metastatic | 692 | 35.3 | 4,694 | 20.0 |  | 692 | 35.3 | 578 | 28.4 |  |
| Unknown | 501 | 25.5 | 5,410 | 23.0 |  | 501 | 25.5 | 454 | 22.3 |  |
| **Mental health condition type ^c692^** | | | | | | | | | | |
| Serious mental illness ^#^ | 560 | 19.6 | - | - |  | 439 | 16.5 | - | - |  |
| Anxiety | 340 | 11.9 | - | - |  | 306 | 11.5 | - | - |  |
| Depression | 487 | 17.0 | - | - |  | 407 | 15.3 | - | - |  |
| Others | 1473 | 51.5 | - | - |  | 1,503 | 56.6 | - | - |  |
| **Virtual outpatient use** | | | | | | | | | | |
| No use | 1,446 | 50.6 | 17,492 | 54.0 | 0.16 | 1,327 | 50.0 | 1,412 | 53.2 | 0.04 |
| Low use (≤3 use/year) | 771 | 27.0 | 9,199 | 28.4 |  | 726 | 27.3 | 734 | 27.6 |  |
| High use (≥ 4 use/year) | 643 | 22.4 | 5,696 | 17.6 |  | 602 | 22.7 | 509 | 19.2 |  |
| **COVID-19 lockdown** | | | | | | | | | | |
| No | 2,015 | 70.5 | 21,676 | 66.9 | 0.05 | 1,839 | 69.3 | 1,869 | 70.4 | 0.00 |
| Yes | 845 | 29.6 | 10,711 | 33.1 |  | 816 | 30.7 | 786 | 29.6 |  |

** Standardised mean difference.
\# Serious mental illness included schizophrenia spectrum and related disorders and bipolar affective disorders.*
***a*** *Cancer morphology data were available only from the Queensland and Victorian Cancer Registries and were therefore not included in propensity score matching due to the absence of comparable data from New South Wales.*
***b*** *Cancer stage at diagnosis was available only from the New South Wales Cancer Registry and was not included in propensity score matching due to the absence of comparable data from Queensland and Victoria.*
***c*** *Mental health condition types are presented for descriptive purposes only and were not included in propensity score matching because mental health status was the primary exposure variable.*

**Supplementary Table 3. Factors associated with unplanned healthcare utilisation in the sensitivity analysis cohort with mental health comorbidity defined using a 12-month pre-index lookback period, New South Wales, Queensland and Victoria, Australia, 2018–2021.**

| **Characteristics** | **Unplanned ED visits** | **Low acuity unplanned ED visits** | **Unplanned hospitalisations** | **Unplanned hospitalisations due to cancer** |
| --- | --- | --- | --- | --- |
|  | **Adjusted IRR, (95 % CI)** | **Adjusted IRR, (95 % CI)** | **Adjusted IRR, (95 % CI)** | **Adjusted IRR, (95 % CI)** |
| **Mental health comorbidity** | | | | |
| No | 1 | 1 | 1 | 1 |
| Yes | 1.41 (1.32 – 1.51) | 1.43 (1.27 – 1.60) | 1.50 (1.39 – 1.61) | 1.48 (1.35 – 1.63) |
| **Age category** | | | | |
| 18 – 44 years | 1 | 1 | 1 | 1 |
| 45 – 64 years | 0.84 (0.73 – 0.95) | 0.80 (0.64 – 1.01) | 1.13 (0.90 – 1.42) | 0.95 (0.85 – 1.06) |
| 65 – 74 years | 0.78 (0.68 – 0.89) | 0.76 (0.60 – 0.97) | 1.01 (0.80 – 1.26) | 0.87 (0.76 – 1.00) |
| ≥75 years | 0.72 (0.63 – 0.82) | 0.85 (0.67 – 1.08) | 0.82 (0.65 – 1.03) | 0.85 (0.76 – 0.95) |
| **Gender** | | | | |
| Male | 1 | 1 | 1 | 1 |
| Female | 0.95 (0.88 – 1.01) | 1.01 (0.90 – 1.15) | 1.05 (0.96 – 1.16) | 0.96 (0.92 – 1.01) |
| **Country of birth** | | | | |
| Australia | 1 | 1 | 1 | 1 |
| Other | 0.99 (0.92 – 1.07) | 0.77 (0.67 – 0.88) | 1.11 (1.00 – 1.24) | 1.08 (1.02 – 1.14) |
| **Rurality** | | | | |
| Urban | 1 | 1 | 1 | 1 |
| Rural | 1.14 (1.05 – 1.24) | 1.91 (1.67 – 2.19) | 0.90 (0.83 – 0.98) | 0.93 (0.83 – 1.03) |
| **Socio-economic status** | | | | |
| Q1 (Most disadvantaged) | 1 | 1 | 1 | 1 |
| Q2 | 0.91 (0.82 – 1.01) | 0.86 (0.73 – 1.02) | 1.02 (0.92 – 1.13) | 1.00 (0.88 – 1.14) |
| Q3 | 0.82 (0.74 – 0.90) | 0.67 (0.56 – 0.79) | 0.98 (0.88 – 1.08) | 0.92 (0.80 – 1.04) |
| Q4 | 0.88 (0.79 – 0.98) | 0.80 (0.67 – 0.97) | 1.00 (0.90 – 1.13) | 1.03 (0.89 – 1.20) |
| Q5 (Least disadvantaged) | 0.67 (0.60 – 0.75) | 0.69 (0.56 – 0.83) | 0.80 (0.72 – 0.89) | 0.79 (0.68 – 0.92) |
| **COVID-19 lockdown** | | | | |
| No | 1 | 1 | 1 | 1 |
| Yes | 0.95 (0.87 – 1.03) | 0.89 (0.77 – 1.01) | 0.84 (0.77 – 0.91) | 0.74 (0.66 – 0.82) |
| **Charlson comorbidities, excluding malignancy** | | | | |
| No | 1 | 1 | 1 | 1 |
| Yes | 1.17 (1.08 – 1.28) | 1.14 (0.97 – 1.35) | 1.17 (1.08 – 1.27) | 1.70 (1.51 – 1.93) |
| **Cancer type** | | | | |
| Breast | 1 | 1 | 1 | 1 |
| Digestive organs including colorectal | 1.65 (1.41 – 1.93) | 1.34 (1.05 – 1.71) | 2.25 (1.85 – 2.74) | 3.77 (2.64 – 5.39) |
| Lung | 1.73 (1.47 – 2.02) | 1.19 (0.92 – 1.53) | 2.41 (1.98 – 2.92) | 4.62 (3.26 – 6.54) |
| Prostate | 0.90 (0.55 – 1.47) | 0.72 (0.40 – 1.30) | 1.30 (0.66 – 2.58) | 0.72 (0.29 – 1.82) |
| Other cancers/ Unknown primary site/ill defined | 1.21 (0.95 – 1.54) | 1.11 (0.76 – 1.64) | 1.81 (1.36 -2.39) | 2.00 (1.08 – 3.72) |
| **Virtual care use** | | | | |
| No use | 1 | 1 | 1 | 1 |
| Low use (≤3 use/year) | 1.33 (1.22 – 1.45) | 1.37 (1.18 – 1.59) | 1.28 (1.18 – 1.40) | 1.18 (1.06 – 1.33) |
| High use (≥ 4 use/year) | 1.89 (1.73 – 2.07) | 2.01 (1.73 – 2.32) | 1.79 (1.63 – 1.96) | 1.54 (1.36 – 1.75) |
